# The microbiota-related coinfections in COVID-19 patients: a real challenge

**DOI:** 10.1186/s43088-021-00134-7

**Published:** 2021-08-21

**Authors:** Ranjan K. Mohapatra, Kuldeep Dhama, Snehasish Mishra, Ashish K. Sarangi, Venkataramana Kandi, Ruchi Tiwari, Lucia Pintilie

**Affiliations:** 1 Department of Chemistry, Government College of Engineering, Keonjhar, Odisha 758002 India; 2 Division of Pathology, ICAR-Indian Veterinary Research Institute, Izatnagar, Bareilly, Uttar Pradesh 243122 India; 3 School of Biotechnology, KIIT University, Bhubaneswar, Odisha 751024 India; 4 Department of Chemistry, School of Applied Sciences, Centurion University of Technology and Management, Odisha, India; 5 Department of Microbiology, Prathima Institute of Medical Sciences, Karimnagar, Telangana India; 6 Department of Veterinary Microbiology and Immunology, College of Veterinary Sciences, Uttar Pradesh Pandit Deen Dayal Upadhyaya Pashu Chikitsa Vigyan Vishwavidyalaya Evam Go Anusandhan Sansthan (DUVASU), Mathura, 281001 India; 7 Department of Synthesis of Bioactive Substances and Pharmaceutical Technologies, National Institute for Chemical and Pharmaceutical Research and Development, Bucharest, Romania

**Keywords:** COVID-19, SARS-CoV-2, Microbiota, Resident microflora, Black fungus, Coinfection

## Abstract

**Background:**

The severe acute respiratory syndrome coronavirus 2 (SARS-CoV-2), the cause of ongoing global pandemic of coronavirus disease 2019 (COVID-19), has infected millions of people around the world, especially the elderly and immunocompromised individuals. The infection transmission rate is considered more rapid than other deadly pandemics and severe epidemics encountered earlier, such as Ebola, Zika, Influenza, Marburg, SARS, and MERS. The public health situation therefore is really at a challenging crossroads.

**Main body:**

The internal and external and resident microbiota community is crucial in human health and is essential for immune responses. This community tends to be altered due to pathogenic infections which would lead to severity of the disease as it progresses. Few of these resident microflora become negatively active during infectious diseases leading to coinfection, especially the opportunistic pathogens. Once such a condition sets in, it is difficult to diagnose, treat, and manage COVID-19 in a patient.

**Conclusion:**

This review highlights the various reported possible coinfections that arise in COVID-19 patients vis-à-vis other serious pathological conditions. The local immunity in lungs, nasal passages, oral cavity, and salivary glands are involved with different aspects of COVID-19 transmission and pathology. Also, the role of adaptive immune system is discussed at the site of infection to control the infection along with the proinflammatory cytokine therapy.

## Background

The ongoing human-to-human transmitted coronavirus disease 2019 (COVID-19), caused by the latest coronavirus strain (severe acute respiratory syndrome coronavirus 2; SARS-CoV-2), is debatably believed to have originated from pangolins and/or bats that has spread rapidly worldwide [1, 2]. SARS-CoV-2 infection as the ongoing pandemic, resulting in increased number of COVID cases with the current second wave, is a serious global health concern, especially for the immunocompromised and the elderly. Viral infections like Ebola, Zika, Influenza, SARS-CoV, MERS-CoV-2, and Marburg have been infecting millions of humans, animals, and birds equally either as a seasonal epidemic or as a pandemic and a global health disaster [3]. These spread through person-to-person contact with body fluids, and there is no effective therapeutics to treat them being viral entities. Like other CoVs, SARS-CoV-2 possesses membrane glycoprotein, spike protein, nucleocapsid protein, small membrane protein, and hemagglutinin esterase [4, 5]. The glycoprotein spikes present on the outer surface of the virus are mostly responsible for its attachment and entry to the host cell [6]. SARS-CoV and MERS-CoV recognize exopeptidases as the key receptor in case of humans [7], while aminopeptidases or carbohydrates in others. MERS-CoV binds to DPP4 while SARS-CoV and SARS-CoV-2 bind to angiotensin-converting enzyme 2 (ACE2) as a key receptor [7, 8]. The virus spike (S)-protein may bind to ACE2 receptors present on various human cells to initiate its entry into the human host cells [6, 9]. ACE2 is found in human cells like in lung alveolar epithelial cells [10]. However, understanding the dynamics of SARS-CoV-2 in humans and its impact is at its infancy [11]. This viral infection reportedly has caused pulmonary, cardiac, renal, circulatory, gastrointestinal, and neurological fatal tissue damage in patients.

**Fig. 1:**
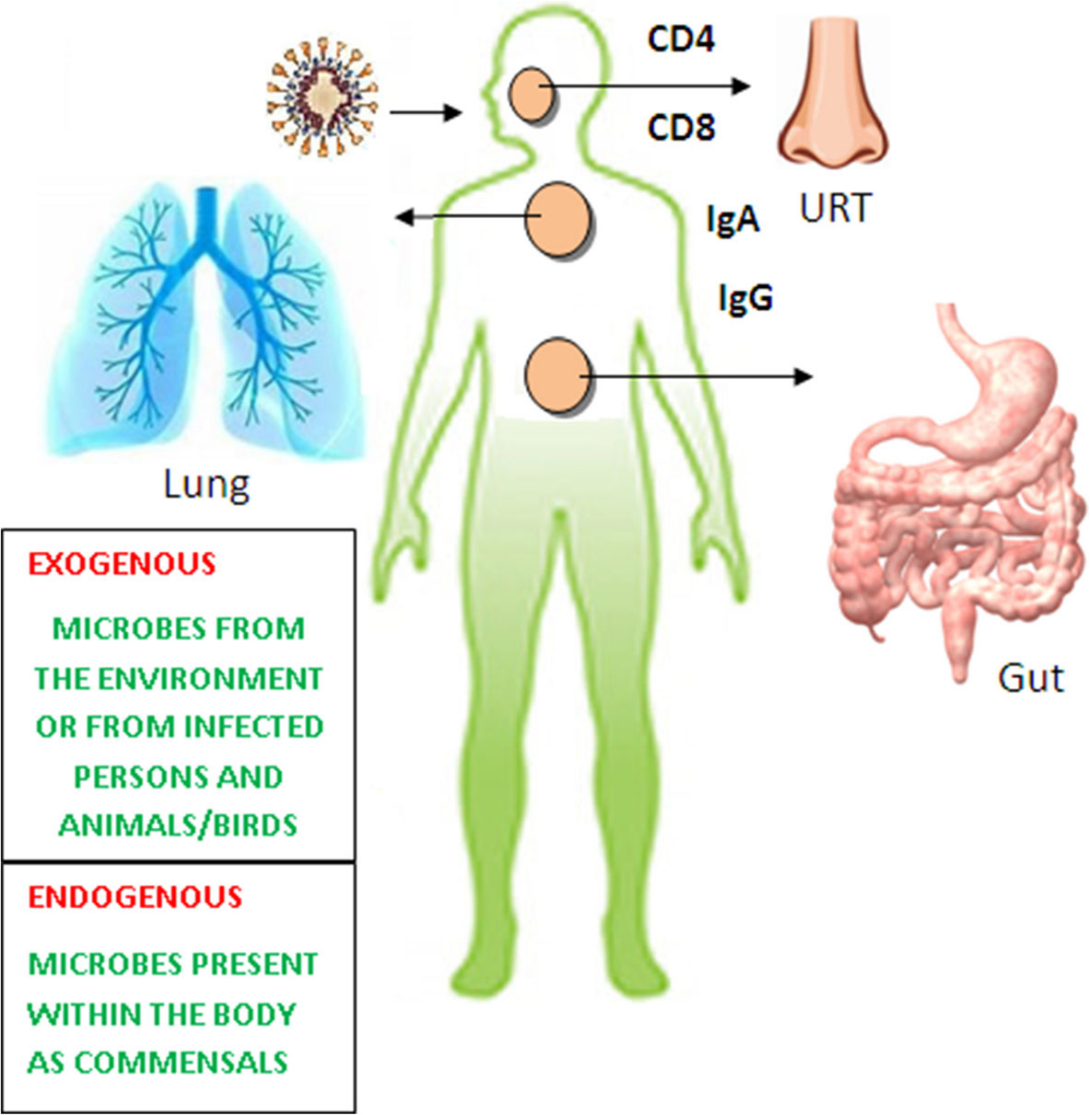
Coinfections observed in COVID-19 cases

The most common symptoms for COVID-19 are cold, fever, and cough, followed by pneumonia. Apart from these respiratory affections, the virus may further affect the heart, kidneys, and the nervous system. It may cause severe complications among the immunocompromised, including those having diabetes and cardiovascular disorders [12, 13]. SARS-CoV-2 is mainly transmitted through the respiratory droplets from the infected, and also through direct/indirect contacts (i.e., contaminated object/surface/fomite) and fecal-oral route [14]. The WHO till date reported millions of deaths due to this novel virus. Respiratory viral infections lead to secondary coinfections and increase the disease severity and mortality outcomes [15]. Microbial coinfection also increases the risk of disease severity in humans [16]. The mechanism of virus interactions with other microbes is still unclear. It is very essential to study the source and the mechanism infection of the coinfecting pathogens. In 1918 influenza outbreak, Morens et al. [17] suggested that most fatalities occurred due to a subsequent coinfection by *Streptococcus pneumonia*. Bacterial coinfection was also associated with the 2009 H1N1 influenza pandemic [18, 19]. There are reports on the bacterial and fungal coinfections (Fig. 1) in COVID-19 pandemic, and the related fatalities [20, 21]. The state-of-art mNGS technique helps to investigate and identify the novel pathogen directly from clinical samples [22] which has confirmed the presence of an elevated level of oral and upper respiratory commensal bacteria [23]. An oral-lung aspiration axis may be a key factor for many infectious diseases [24].

## Main text

### Viral coinfection

Coinfection is commonly encountered in respiratory diseases [25] which influences disease prognosis and treatment (Table 1). Viral coinfections in COVID-19 patients have been reported globally, and are critical during early misdiagnosis [50]. Possibly due to their immunity status, the middle-aged and the elderly are more prone to viral coinfection [26]. However, it may not be true, healthy people may also be coinfected [27]. Majority of COVID-19 patients coinfected with other viruses have been reported to be around 30–60 years old [51]. An in vitro study by Lin and coworkers at Shenzhen Third People’s Hospital confirmed 3.2% viral coinfection, and at least two viruses were detected in 2.2% of those patients [52]. A study in Wuhan confirmed 5.8% coinfections with other coronavirus, hRV, and influenza (H3N2) [27]. Additional pathogens in 20.7% COVID-19-positive specimens were reported from Northern California, predominantly by RSV, entero-/rhinovirus, and non-SARS-CoV-2 CoV [28]. Due to the infectivity nature of SARS-CoV-2, respiratory viruses like hepatitis virus [29] and HIV [30] coinfections were noticed along with simultaneous detection of common respiratory viruses like RSV, hMPV, hRV, PIV2, and HKU1. Also, *C. pneumoniae*, parainfluenza 3, influenza A, *M. pneumoniae*, rhinovirus, and non-SARS-CoV-2 CoV are common coinfections [26].

**Table 1:** Microbial groups reportedly active in coinfection of COVID-19 patients

Microbial group	Microbe(s)	Origin	Endogenous/exogenous	Found in	References
Virus	*C. pneumoniae*, parainfluenza 3, influenza A, *M. pneumoniae*, rhinovirus, non-SARS-CoV-2	Environment/birds/animals	Exogenous	Upper/lower respiratory tract	[26]
	Other coronavirus, hRV, influenza (H3N2)	Environment/birds/animals	Exogenous	Upper/lower respiratory tract, intestine	[27]
	RSV, entero/rhinovirus, non-SARS-CoV-2	Environment/birds/animals	Exogenous	Upper/lower respiratory tract, intestine	[28]
	Hepatitis virus	Human	Endogenous/exogenous	Blood, tissues, body secretions	[29]
	HIV	Human	Endogenous/exogenous	Blood, tissues, body secretions	[30]
	hMPV, hRV, PIV2, HKU1	Human, animals	Exogenous	Upper/lower respiratory tract	[29, 30]
Bacteria	*Veillonella*, *Capnocytophaga*,	Human	Endogenous	Oral cavity	[23, 31–33]
	*Neisseria*, *Streptococcus pneumoniae*, *Corynebacterium*, *Leptotrichia*, *Prevotella*, *Fusobacterium periodonticum*	Human	Endogenous	Oral cavity	[34, 35]
	*Pseudomonas aeruginosa*, *Streptococcus pneumoniae*, *Fusobacterium periodonticum*, *Veillonella*, *Prevotella, Capnocytophaga*	Human	Endogenous/exogenous	Upper/lower respiratory tract	[23, 31–33]
	*Staphylococcus aureus*, *Haemophilus influenza*, *Escherichia coli*	Human/environment	Endogenous/exogenous	Respiratory tract, skin, and intestines	[36]
	*Coprobacillus*, *Clostridium hathewayi*, *C. ramosum*	Human, environment	Endogenous/exogenous	Gut	[37]
	*Clostridium*, *Veillonella*, *Actinomyces*, *Streptococcus pneumoniae*, *Rothia*	Human, environment	Endogenous/exogenous	Gut	[25, 37–40]
	*Mycobacterium tuberculosis*	Human, environment	Endogenous/exogenous	Lower respiratory tract, other organs of the body	[41–43]
Fungi	*Candida tropicalis*, *C. albicans*, *C. glabrata*, *C. dubliniensis, C. krusei*	Human	Endogenous	Upper/lower respiratory tract, intestine	[44]
	*Aspergillus fumigatus*	Human, environment	Endogenous/exogenous	Upper/lower respiratory tract	[45]
	*Aspergillus fumigatus*, *Rhizopus oryzae, Absidia mucor*	Human, environment	Endogenous/exogenous	Respiratory tract, other organs of the body	[46]
	Black fungus (*Rhizomucor species*, *Syncephalastrum species*, *Cunninghamella bertholletiae, Apophysomyces, Lichtheimia*, *Saksenaea*, *Rhizomucor*)	Environment	Majorly exogenous	Respiratory tract, eye, broken skin and its appendages, sinuses, and brain	[47–49]

### Viral coinfection and immune response

The respiratory viral infections normally affect the airways and lungs. Among all, the Influenza virus is responsible for causing frequent seasonal viral infections [3]. Other viruses responsible for respiratory infections include coronavirus, human adenovirus, rhinovirus, enterovirus, parainfluenza virus, and human metapneumovirus. Viral coinfection influences the prognosis and treatment of COVID-19, and such patients need higher level of care [53]. Development of such coinfections affects the host immune response, especially in the immunocompromised and elderly people [54]. Reportedly, the patients having hepatitis C virus and HIV infections more likely lead to drug-induced liver injury (DILI) [55]. COVID-19 infection may cause liver damage [56]. As coinfection causes serious damage to immunity [57], so patient’s condition may be more serious, the treatment could be more complicated, and the treatment cycle may be longer [58]. Patients that are coinfected with SARS-CoV-2 and HIV had a longer disease progression attributed to the slower specific antibody generation [59]. Genome sequencing confirms that SARS-CoV-2 is 79.5% identical with SARS-CoV [5].

### Rationale of viral coinfection

Viral coinfection increases the CRP and PCT levels, damaging the immunity and the airway [60, 61]. Viral coinfections arise as the airway epithelium is destroyed by SARS-CoV-2 virus. COVID-19 could cause immune system disorders leading to a possibility of coinfection by other viruses [62]. Coinfection mechanism is unclear in COVID-19 patients due to very little available information about the virus kinetics. Coinfection rate in COVID-19 with other viruses is reportedly not very high [63]. Prevention and control of infection is suggested in COVID-19 patients to avoid coinfection [64]. Social distancing is arguably the best prevention in the spread of infection [65–67]. Isolating the patients during treatment in a clinical setting is suggested to understand the transmission risk of the infection [67]. Patients with HIV infection history are more likely to encounter COVID-19 coinfection due to their reduced specific antibody responses [68].

### Bacterial and fungal coinfection

Bacterial coinfection is a worrying problem in the COVID-19 management and also is the major cause of morbidity and mortality in other respiratory infections [69]. However, the rate of coinfection in COVID-19 patients is relatively low possibly due to limited available studies. Contou et al. [70] reported 28% bacterial coinfection in French ICU patients with SARS-CoV-2, mostly related to *Haemophilus influenzae*, *Staphylococcus aureus*, *Streptococcus pneumonia*, and bacteria of Enterobacteriaceae family. A recent meta-analysis also confirmed bacterial and viral coinfections in COVID-19 patients [71]. Bacterial coinfection is reportedly more (14%) in COVID-19 patients in the ICU [72]. Calcagno and coworkers reported coinfections with other respiratory pathogens such as *Staphylococcus aureus*, *Moraxella catarrhalis*, *Haemophilus influenzae*, *Streptococcus agalactiae*, *Enterobacter cloacae*, *Klebsiella pneumoniae*, and *Escherichia coli* in COVID-19 patients [73]. A study on 989 COVID-19 patients showed nosocomial superinfections [74]. A total of 51 hospital-acquired bacterial superinfections by *Escherichia coli* and *Pseudomonas aeruginosa* along with *S. pneumoniae*, *S. aureus* and *Klebsiella pneumoniae* were diagnosed. Also, *mycobacterium tuberculosis* coinfection was observed in COVID-19 patients [41–43], although such coinfections reportedly do not frequently occur. Mohamed and coworkers reported multi-triazole resistant *Aspergillus fumigates* coinfection in respiratory samples and suggested that early diagnosis would help to understand the antifungal therapy to improve the diseases condition [45]. In a case report, Pal and coworkers found *Streptococcus pneumoniae* coinfection in SARS-CoV-2-infected patients [75]. *S. pneumoniae*, *M. pneumoniae*, *L. pneumoniae*, and *C. pneumoniae* coinfections are also observed in COVID-19 patients and suggested for combination therapy with non-anti-SARS-CoV-2 agents [76]. In a multicentre cohort study, Russell and his group reported 70.6% secondary nosocomial infections in COVID-19 cases during the first wave [36]. *Staphylococcus aureus*, *Haemophilus influenzae*, and *Escherichia coli* (Enterobacteriaceae) were the most commonly encountered pathogens as diagnosed within two days post hospitalization.

### Human saliva and COVID-19

Human saliva constituting 94–99% water content, produced by the salivary gland, is important in food digestion, oral mucosa lubrication, cleaning, and preservation of oral cavity. It also contains food particles, oral microbes and their metabolites, serum elements, white blood cells, and exfoliated epithelial cells. Although more than 700 microbial species are detected in it, saliva prevents overgrowth of specific pathogens and serves as a gatekeeper (the first level of defense), and prevents them from spreading to the respiratory and gastrointestinal tracts [65]. Also, it is crucial in preventing viral infection [77]. SARS-CoV-2 may enter human saliva through the lower and upper respiratory tract droplet nuclei. It may enter the mouth through the blood from gingival crevicular fluid, and through salivary ducts from infected salivary gland [78].

A previous study on SARS-CoV confirmed infection of epithelial cells of salivary gland having elevated angiotensin-converting enzyme 2 (ACE2) expressions [79]. Moreover, ACE-2 expression in minor salivary glands was found to be more than in lungs. Before the onset of lung lesions, SARS-CoV RNA may be found in saliva samples. Live virus may be cultured in saliva samples. Thus, salivary gland is a significant virus reservoir. It suggests that SARS-CoV-2 spreads through contaminated saliva for asymptomatic infections [80].

### Oral bacterial microbiota

Significant number of viral, bacterial, and fungal coinfections in COVID-19 originating from the oral cavity has been observed, similar to other pandemics. Oral pathogens like *Veillonella* and *Capnocytophaga* were confirmed by mNGS in bronchoalveolar lavage fluid (BALF) of COVID-19 cases [31]. A higher nasal virus load in the throat has been reported [81]. Oral cavity houses the second largest microbiota containing bacteria, viruses, fungi, and archaea in human body [82]. Major bacterial genera in human oral cavity are *Neisseria*, *Prevotella*, *Streptococcus*, *Corynebacterium*, *Fusobacterium*, *Leptotrichia*, *Veillonella*, and *Capnocytophaga* [34]. Many such pathogens may colonize the respiratory tract of healthy individuals asymptomatically [83]. Thus, oral microbiome regulates mucosal immunity and affects pathogenicity [84].

### Lung microbiota

In COVID-19, the virus infects epithelial cells of the upper respiratory tract (URT) like the nasal passages and throat, and lungs (bronchi and lung alveoli). The local immunity in lungs, nasal passages, oral cavity, and salivary glands are involved with different aspects of SARS-CoV-2 transmission and pathology. The lung microbiota community is another complex variety and found in lower respiratory track (LRT) like the epithelial and mucous layers. There is a relationship between the microbial community in lungs and the oral cavity [85]. Under normal conditions, the microbiota from oral cavity migrates as an important source of lungs microbiota [86]. Human lungs contain *Pseudomonas*, *Streptococcus*, *Prevotella*, *Fusobacterium*, *Veillonella*, and *Capnocytophaga* that is found in oral cavity as well [23, 32, 33]. Sometimes, potentially harmful bacteria responsible for respiratory disorders like *S. pneumonia*, *H. influenza*, and *M. catarrhalis* are also found in respiratory specimens. Further, the fungal genera include *Candida*, *Aspergillus*, *Saccharomyces*, and *Malassezia*. Studies confirm that lung microbiota is quite similar to those in the oropharynx and nasopharynx [44].

Reports mention that 72% COVID-19 patients received antimicrobial therapy to treat fungal and bacterial coinfections [87, 88], although the pathogenesis was unclear. As active microbiota of the oral cavity is found in the BALF of COVID-19 patients, it could be a natural reservoir of opportunistic pathogens in COVID-19 patients. Metagenomic sequencing confirms that the nasopharyngeal *Fusobacterium periodonticum* population in SARS-CoV-2 patients varied with the duration of the infection and decreased significantly beyond 3 days [35].

### Intestinal microbiota

Ingestion is a frequent mode of pathogen transmission; gastrointestinal infection is common among the pediatric age group attributable to their playing habits. Environmental microbes are accidentally ingested by both humans and animals, although most of them do not necessarily result in infection. This could be attributed to the unfriendly acidic environment in the stomach and the various proteolytic enzymes in the alimentary system. The mucus lining, the peristaltic movements of the intestinal villi, the secretory immunoglobulins, the local immune defence mechanisms like mucosa-associated lymphoid tissue (MALT) and gut-associated lymphoid tissue (GALT) also aid in the first line of defense. However, microbes like bacteria and viruses occasionally succeed in causing gastrointestinal disorders. SARS-CoV-2 is transmitted through the respiratory route and not much is known about the presence/survival of it in the intestine and transmission through the fecal-oral route. The consequence of SARS-CoV-2 infection in the gastrointestinal tracts is unclear [89, 90]. Studies report the potential of SARS-CoV-2 in the faeces of infected persons and its possible faecal-oral transmission. This is supported by several reports hinting at diarrhoea as a clinical presentation among a quarter of patients infected by the pandemic. Common gastrointestinal symptoms include nausea, diarrhea, vomiting, and abdominal pain [91–96], that persist in throat swabs in SARS-CoV-2 convalescence with diminished respiratory symptoms.

Intestinal microbiota influence pulmonary diseases [97]. Studies demonstrate that respiratory viral infection may disturb intestinal microbiota [94, 98–101]. Gut microbiota may downregulate the ACE2 expression with virus load in COVID-19 cases [37]. Results demonstrate that SARS-CoV-2, which attaches to the ACE2 receptors and transmembrane serine protease 2, could infect intestinal epithelial cells. These cells exhibited receptors to bind to the virus as do the respiratory epithelial cells and other cells. Thus, SARS-CoV-2 efficiently adheres to intestinal epithelial cells, causes inflammation, and could initiate infection via the gastrointestinal tract [102]. SARS-CoV-2 may cause local inflammation in the gut and could lead to coinfection taking advantage of suppressed immune system resulting in severe infection, especially among the elderly. The novel virus caused dysbiosis of the gut microbiome potentially facilitating its invasion and survival. Disturbed normal gut microbiome predisposes the patients to secondary microbial infections and dissemination of virus to other body parts [103, 104]. The susceptibility to SARS-CoV-2 in patients with irritable bowel disease (IBD) and other luminal diseases has been reported. This could be supporting evidence that a healthy gut with normal microbiome prevents potential SARS-CoV-2 spread by faecal-oral route.

Indians have a comparatively healthy gut attributable to their eating habits, ensuring the existence of health-benefiting microbes [105]. There is an increased belief that probiotics help in managing and prognosis of COVID-19. Probiotics could prevent excessive immune response (cytokine storm), reduce inflammation and prevent virus multiplication and invasion [106, 107]. COVID-19 remains mild and becomes self-limiting in healthy individuals with a robust immunity. SARS-CoV-2 infection increases in severity causing complications and death with a compromised immunity and other debilitating conditions like diabetes and increased age. This supports the argument that the immunity status of individuals plays a key role in COVID-19 disease prognosis. As gut microflora influences the immune system, disturbances of the gut microbiome may predispose people to COVID-19 via intestinal invasion. Also, there could be an increased likelihood of secondary microbial coinfections as observed in HIV infection and acquired immunodeficiency syndrome (AIDS) wherein the patients suffer from serious intestinal parasitic infections involving opportunistic microbes [108].

A comparison between the gut microbiome of COVID-19, H1N1 influenza patients, and the healthy controls indicated that opportunistic bacterial (*Clostridium*, *Veillonella*, *Actinomyces*, *Streptococcus* and *Rothia*) and fungal (*Candida* and *Aspergillus*) pathogens replaced beneficial microbes/commensals like *Proteobacteria*, *Bacteroides*, *Actinobacteria*, *Blautia*, *Romboutsia*, *Collinsella*, and *Bifidobacterium* in COVID-19 patients. Also, unique bacterial species were noticed in COVID-19 patients that could be infection indicators in SARS-CoV-2 [25, 37–40]. *Coprobacillus*, *Clostridium hathewayi*, and *C. ramosum* have been reported to be associated in severe COVID-19. *Bacteroides* sp. downregulated ACE2 expression in the murine gut and were correlated inversely with the SARS-CoV-2 load [37, 109].

**Fig. 2:**
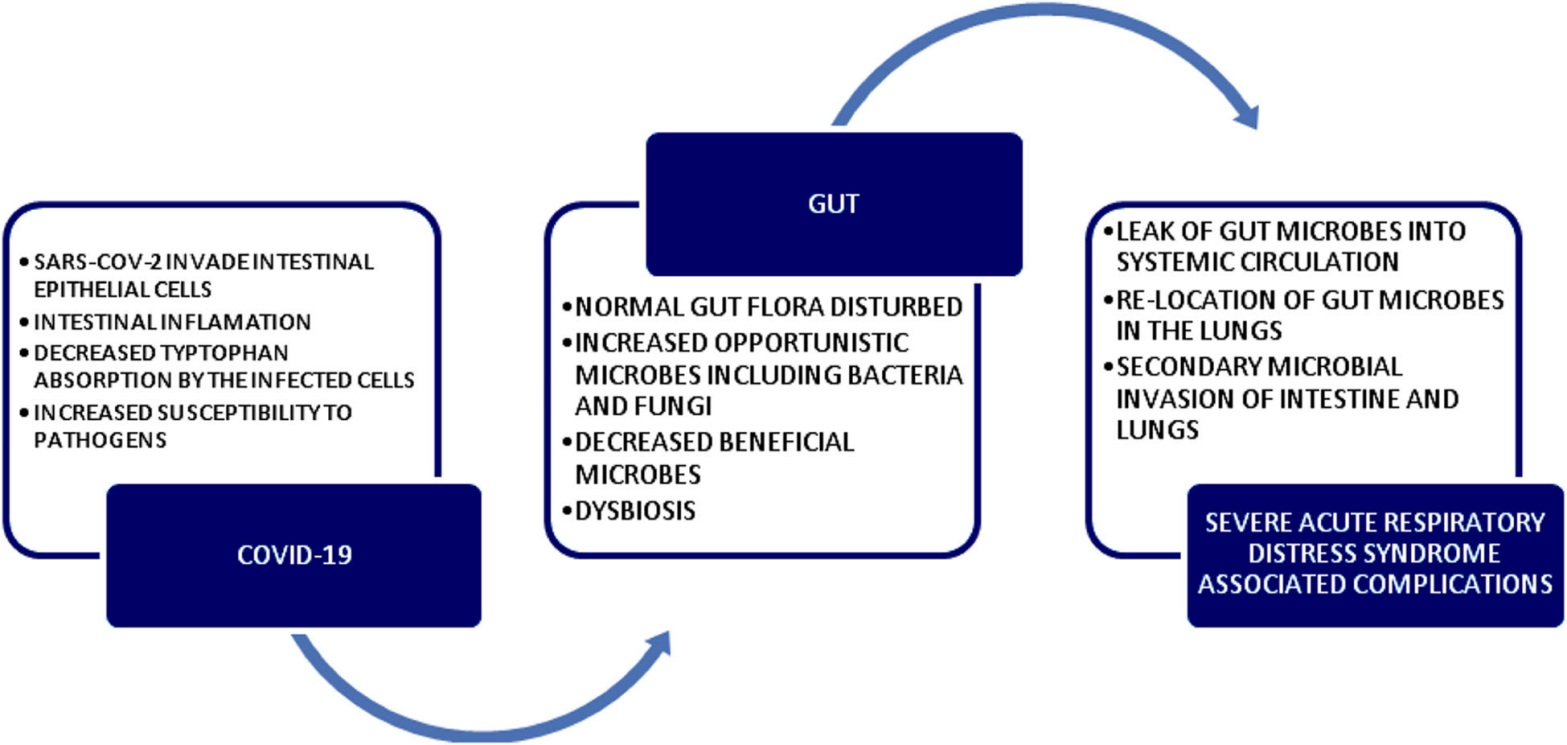
Gut microbiota and COVID-19

An assessment of the pulmonary and intestinal microflora that influences the prognostic to determine the clinical outcome of COVID-19 patients and therapeutics has been reported. The ACE-2 on intestinal epithelial cells facilitates absorption of tryptophan, an antimicrobial peptide. As SARS-CoV-2 attaches to the ACE-2 receptors on the epithelial cells causing reduced absorption of tryptophan and increased survival of microbes, it predisposes COVID-19 patients to severe complications and secondary microbial coinfections [110]. Fecal shedding of SARS-CoV-2 in convalescing patients and dysbiosis of gut microbiome even after a month has been reported. Study proposed the screening of fecal specimen for SARS-CoV-2 before fecal microbiota transplantation procedures [111]. It is important that the normal pulmonary and intestinal microflora is maintained in equilibrium as there is a harmonious relationship between the gut microbiome and the respiratory health [112]. Because COVID-19 disturbs the gut and the airway microbiome, it predisposes the patients to gastrointestinal and respiratory complications (Fig. 2).

### Proinflammatory cytokine therapy

The fundamental components (B cells, CD4+ T cells, and CD8+ T cells) of the adaptive immune system are important to control the viral infections. These play different roles in different viral infections and it is very essential to understand the COVID-19-associated mechanism. Adaptive immune system should act at the site of infection to control an infection [113]. Although the mechanism of viral entry is still ambiguous [114, 115], severe COVID-19 patients demonstrated an over-reactive immune response leading to cytokine storm and developing acute respiratory distress syndrome (ARDS) [114]. ARDS leads to other complications like secondary bacterial infections and lung fibrosis. In severe cases, host-directed immunotherapy is an adjunct therapy that could reduce inflammation and related lung damage and prevent ICU hospitalization. Cytokine storm syndrome is a major cause of mortality associated with hospitalized COVID-19 patients [116]. Cytokine storm from several viral infections is well-known to be involved in enhancing immunopathology of the disease [117]. A high level of inflammatory cytokine (IL-6) was reported during early pandemic days in COVID-19 patients, with more than 80 pg/mL IL-6 levels, a good indicator of respiratory failure and death [118]. Targeting IL-1 (another pro-inflammatory cytokine) could be a successful strategy to improve survival in COVID-19 patients [119, 120]. Cavalli and coworkers compared the effectiveness of IL-1 and IL-6 inhibition in treating COVID-19 cytokine storm syndrome [121]. In a significant number of mortality cases, SARS-CoV-2 was associated with extensive multiorgan inflammation suggesting a maladaptive immune response, resulting in continuous neutrophil activation and organ damage [117].

Anti-inflammatory therapy is being explored in morbidity and mortality reduction. Immunosuppressive therapies like cytokine blockade and JAK inhibition is also suggested [122]. The first therapy that reduced mortality was dexamethasone. Recent studies have shown the benefit of tocilizumab in critically ill patients, and baricitinib in hospitalized patients providing substantial evidence that COVID-19 patients benefit from immunosuppressive therapies [123]. Glucocorticoid therapy may also be beneficial for COVID-19 treatment [124]. Cytokine-targeted treatment by anakinra was promising in saving lives in COVID-19 cases, although randomized controlled trails results are awaited [124, 125].

### Animal models in coinfection study

Various microbial coinfections are a common occurrence in several epidemics and pandemics including three lethal CoVs witnessed in last two decades. It is very essential to understand the pathogenesis and nosocomial management of SARS-CoV-2 and related coinfections. Mouse model is widely used for different viral pathogenesis investigations due to its small size and easy low cost of operation. Studies to determine the role of immune effectors in the CoV infection report the use of immunocompromized mice [126].

The current SARS-CoV-2 mouse model may be critical in line with considering the use of MHV to study its biological mechanisms [127], using gene editing technology to understand mouse genes (ACE2 and TMPRSS2) related to viral binding and entry [128, 129], or transfer of human ACE2 for direct infection [130], and using wild SARS-CoV-2 virus to establish a mouse model for significant clinical phenotype [131]. To understand the coinfection mechanism by inoculating other pathogens, a coinfection mouse model may be recommended [132]. Furthermore, small animals like human ACE2 transgenic mice, wild-type mice, Syrian hamsters, and large animals such as ferrets, cats, Rhesus macaques, and Cynomolgus macaques may contribute significantly as animal models to evaluate vaccines and drugs against SARS-CoV-2 [133, 134].

### Mucormycosis

Mucormycosis, also known as black fungus or zygomycosis, is found in the environment and is caused by a group of molds called mucormycetes that mainly affect the sinuses or the lungs of people with reduced immunity [135]. It is a rare *albeit* deadly fungal infection and is now detected in COVID-19 patients in India too. Many Indian states have reported such infections among the COVID-19 patients. Once a person is infected, this opportunistic pathogenic fungus manifests in the skin or could affect the brain or lungs. As per the *Centre for Disease Control* and Prevention (CDC) of the USA, it may be rhinocerebral mucormycosis (sinus and brain), pulmonary mucormycosis (lung), gastrointestinal mucormycosis (gut and intestines), cutaneous mucormycosis (skin), and disseminated mucormycosis (in people having other medical conditions). Usually developing in 10–14 days post-hospitalization, the infection spreads through the bloodstream to other body parts. The patients may be treated with Amphotericin B (an antifungal), and surgery may be required in some cases.

The symptoms are pain and redness around the eyes or nose, blurred or double vision, loosening of teeth, toothache, blackish/bloody discharge from nose, bloody vomits, swelling in cheekbones, skin lesion, chest pain, fever, headache, dyspnea, coughing, and may also alter mental status [135, 136]. This infection is observed in the convalescing COVID-19 patients having issues related to diabetes, prolonged ICU stay, prolonged medical oxygen use, high blood sugars, chronic kidney disease, HIV/AIDS, hematological malignancies, solid organ transplant, etc. [137] Such infections spread due to the rampant misuse or overuse of steroids, monoclonal antibodies, and broad-spectrum antibiotics during COVID-19 treatment [47]. As India has second largest diabetic population with around 70% are uncontrolled cases, such coinfection has become more common here [135]. Hence, higher mortality rate (~ 87%) is observed these days as compared to earlier reports (~ 50%) during non-COVID times [48, 138].

Garg and coworkers [48] reported a COVID-19-associated pulmonary mucormycosis in a 55-year-old COVID-19 patient with diabetes, end-stage kidney disease. With 5 g of liposomal amphotericin B treatment, the patient was discharged from the hospital after 54 days. They also analyzed seven other cases of COVID-19-associated mucormycosis. According to them, diabetes mellitus was the most common risk factor. The incidence of acute invasive fungal rhinosinusitis is prominent in post-COVID-19 patients especially in the immunocompromised [46], the most common infecting organisms being *Aspergillus fumigatus*, *Rhizopus oryzae*, and *Absidia mucor*.

In India, along with black fungus, white and yellow fungus infections detected during endoscopy proved fatal in COVID-19 patients [139]. While mucormycosis relates to black fungus, however the latter are referred to as aspergillosis, candidiasis, and cryptococcosis. All such fungal infections were observed in immunocompromised COVID-19 patients by invading the immune system leading to dysregulation and reduced numbers of T lymphocytes, CD4+T, and CD8+T cells [46]. Physicians need to be careful about the possibility of such secondary invasive fungal infections in COVID-19 patients during and after the onset of the disease [49].

## Conclusions

The internal and external resident microbiota is crucial in human health and is essential for immune responses. The microbial coinfection increases the risk of disease severity in humans. However, their mechanism of interaction with the infecting virus with other pathogens is still unclear. It is very essential to study the source and the mechanism of the coinfecting pathogens. This will help in early diagnosis and to understand the antimicrobial and antifungal therapy to effectively treat the disease. The use of health microbiobata, probiotics, and other health promoting regimens need to be explored to counter coinfections during COVID-19 pandemic. Experimental therapy to support the treatment outcomes and prevention of the consequences of respiratory coinfection is imminent. This review has attempted to summarize previous studies describing the viral, bacterial, and fungal pathogens involved in COVID-19 coinfections, and it also discusses the role of adaptive immune system at the site of infection to control the infection along with the proinflammatory cytokine therapy.

## Data Availability

Not applicable.

## Abbreviations

CoV: Coronavirus
COVID-19: Coronavirus disease 2019
SARS-CoV-2: Severe acute respiratory syndrome coronavirus 2
RSV: Respiratory syncytial virus
mNGS: Metagenomic next-generation sequencing
BALF: Bronchoalveolar lavage fluid
PIV2: Parainfluenza virus type 2
*M. pneumoniae*: *Mycoplasma pneumoniae*
*C. pneumoniae*: *Chlamydia pneumoniae*
DILI: Drug-induced liver injury
CRP: C-reactive protein
PCT: Procalcitonin
MALT: Mucosa associated lymphoid tissue
GALT: Gut-associated lymphoid tissue
MHV: Mouse hepatitis virus
ARDS: Acute respiratory distress syndrome
